# Differential Effects of Dorsal and Ventral Medial Prefrontal Cortex Inactivation during Natural Reward Seeking, Extinction, and Cue-Induced Reinstatement

**DOI:** 10.1523/ENEURO.0296-19.2019

**Published:** 2019-09-25

**Authors:** Jessica P. Caballero, Garrett B. Scarpa, Luke Remage-Healey, David E. Moorman

**Affiliations:** 1 Neuroscience and Behavior Graduate Program; 2 Department of Psychological and Brain Sciences, University of Massachusetts Amherst, Amherst, MA 01003

**Keywords:** frontal, infralimbic, learning, prelimbic, self-administration, sucrose

## Abstract

Rodent dorsal medial prefrontal cortex (mPFC), typically prelimbic cortex, is often described as promoting actions such as reward seeking, whereas ventral mPFC, typically infralimbic cortex, is thought to promote response inhibition. However, both dorsal and ventral mPFC are necessary for both expression and suppression of different behaviors, and each region may contribute to different functions depending on the specifics of the behavior tested. To better understand the roles of dorsal and ventral mPFC in motivated behavior we pharmacologically inactivated each area during operant fixed ratio 1 (FR1) seeking for a natural reward (sucrose), extinction, cue-induced reinstatement, and progressive ratio (PR) sucrose seeking in male Long–Evans rats. Bilateral inactivation of dorsal mPFC, but not ventral mPFC increased reward seeking during FR1. Inactivation of both dorsal and ventral mPFC decreased seeking during extinction. Bilateral inactivation of ventral mPFC, but not dorsal mPFC decreased reward seeking during cue-induced reinstatement. No effect of inactivation was found during PR. Our data contrast sharply with observations seen during drug seeking and fear conditioning, indicating that previously established roles of dorsal mPFC = going versus ventral mPFC = stopping are not applicable to all motivated behaviors and/or outcomes. Our results indicate that dichotomous functions of dorsal versus ventral mPFC, if they exist, may align better with other models, or may require the development of a new framework in which these multifaceted brain areas play different roles in action control depending on the behavioral context in which they are engaged.

## Significance Statement

Dorsal and ventral medial prefrontal cortex (mPFC) have been proposed to control response execution and inhibition, respectively, in contexts such as drug seeking and fear learning. It is unclear, however, whether these roles are generalizable to all behaviors. We found that these opposing roles were not present during natural reward (sucrose) seeking, in contrast with previous drug seeking and fear conditioning literature. Dorsal and ventral mPFC inactivation did impact multiple aspects of seeking, but not in the bidirectional fashion predicted by a generalized go/stop model. We conclude that, although these brain areas are clearly important in reward seeking, the dichotomous roles proposed previously are not broadly applicable, and mPFC contributions to these and related behaviors should be reconsidered.

## Introduction

The rodent medial prefrontal cortex (mPFC) plays a key role in numerous behaviors and cognitive functions, including action control, emotional regulation, attention, memory, and decision-making, among others (Dalley et al., 2004; Vertes, 2006; Euston et al., 2012; Barker et al., 2014; Cassaday et al., 2014; Moorman et al., 2015; Eichenbaum, 2017; Ko, 2017). Multiple studies have demonstrated that dorsal mPFC (typically prelimbic cortex) and ventral mPFC (typically infralimbic cortex) have opposing roles that facilitate the execution and inhibition, respectively, of behaviors (Peters et al., 2009; Gass and Chandler, 2013; Gourley and Taylor, 2016). These differences have been observed during drug seeking, fear-associated behaviors, and certain studies of natural reward seeking. For example, dorsal mPFC inactivation reduces reinstatement of drugs of abuse such as cocaine or heroin (McFarland and Kalivas, 2001; McLaughlin and See, 2003; Fuchs et al., 2005; LaLumiere and Kalivas, 2008). In contrast, ventral mPFC inactivation increases cocaine seeking during extinction, and activation of ventral mPFC decreases reinstatement of cocaine and other drugs of abuse (LaLumiere and Kalivas, 2008; Peters et al., 2008; Muller Ewald and LaLumiere, 2018). In studies of auditory fear conditioning and extinction, dorsal mPFC inactivation decreases fear expression and ventral mPFC inactivation impairs extinction learning and recall (Maren and Quirk, 2004; Peters et al., 2009; Sierra-Mercado et al., 2011). Dorsal and ventral mPFC may also have opposing roles with respect to natural reward seeking: inactivation of dorsal and ventral mPFC decreases and increases in reward seeking, respectively, in certain behavioral paradigms (Rhodes and Killcross, 2004, 2007; Ishikawa et al., 2008a,b; Sangha et al., 2014; Eddy et al., 2016; Trask et al., 2017).

However, these dorsal versus ventral dichotomies are not always observed, and in some cases opposing functions have been described (Moorman et al., 2015). For example, inhibition of dorsal mPFC in models of cocaine dependence result in increased punishment-resistant drug seeking (Chen et al., 2013). Some studies have found an effect of mPFC manipulation on cocaine, but not natural reward seeking (McFarland and Kalivas, 2001; McGlinchey et al., 2016; Gutman et al., 2017b). In a discriminative stimulus-driven reward seeking task, both dorsal and ventral mPFC neurons fired during reward seeking and extinction, and inactivation of dorsal or ventral mPFC did not result in specific deficits in execution and extinction of reward seeking (Moorman and Aston-Jones, 2015). In a variable interval sucrose seeking task, dorsal mPFC neurons fired during reward delivery and inactivating this region did not alter reward seeking, whereas ventral mPFC neurons fired during reward collection and inactivating the ventral mPFC delayed the collection of reward (Burgos-Robles et al., 2013). Dorsal mPFC has also been associated with goal directed behaviors, attention, or spatial location representation, and ventral mPFC has been associated with habitual behaviors and emotional regulation, among multiple other functions (Killcross and Coutureau, 2003; Dalley et al., 2004; Euston et al., 2012; Smith et al., 2012; Smith and Graybiel, 2013; Cassaday et al., 2014; Gourley and Taylor, 2016).

This diversity of results indicates not only that these areas play complex roles in shaping behavior, but also that there may be differences depending on the tasks used to probe mPFC function. Surprisingly, there has been limited characterization of dorsal versus ventral mPFC contributions to self-initiated instrumental reward seeking and, analogous to described models of drug seeking, extinction and reinstatement. Here we used pharmacological inactivation to characterize the roles of mPFC subregions during these tasks and during a progressive ratio (PR) task to assess motivation. We also performed a preliminary assessment of whether or not individual mPFC hemispheres differentially regulate reward seeking, as seen in other behaviors (Sullivan and Gratton, 2002a,b), and we performed physiological and behavioral controls to verify the effects of our pharmacological manipulations. Despite observing differential effects of dorsal versus ventral mPFC inactivation on reward seeking, our findings do not align with previous observations of go/stop dichotomies. Instead they indicate that these brain areas likely perform multiple functions, befitting their complex integrative nature, and that behavioral context, such as the task employed, dictates the contributions of these regions to the behaviors studied.

## Materials and Methods

### Animals

Male Long–Evans rats (approximately nine weeks old and 275–300 g on arrival; Charles River; *N* = 80) were used in behavioral studies (sucrose self-administration *N* = 40; extinction *N* = 16; cue-induced reinstatement PR *N* = 16; spontaneous locomotion, *N* = 8). Two additional male Long–Evans rats were used for *in vitro* electrophysiology studies (see Whole-cell patch-clamp below for details). All rats were single-housed on a reversed light cycle (7 A.M. on and 7 P.M. off) and allowed free access to food and water. Experiments were conducted during active cycle (lights off). All experiments were conducted in accordance with the National Institute of Health guidelines and the standards of the University of Massachusetts Institutional Animal Care and Use Committee.

### Surgery

Rats were anesthetized with isoflurane in a closed container (5%) and transferred to a stereotaxic frame where they received isoflurane through a nosecone (1.5–2%). Rats were given systemic antibiotic (0.1 ml cefazolin (330 mg/ml)) and analgesic (1mg/kg meloxicam), and incisions were treated with local anesthetic (0.3 ml, 2% lidocane). Bilateral craniotomies were made above the mPFC, and double guide cannulae (26 gauge, Plastics One) were implanted in either dorsal mPFC (+3.0 mm AP; ± 0.6 mm ML; –2.5 mm DV) or ventral mPFC (+3.0 mm AP; ±0.6 mm ML; –4.0 mm DV). Three screws were implanted to secure cannulae with dental cement. Rats were allowed one week to recover following surgery. Rats tested in the spontaneous locomotor assay (see Spontaneous locomotion below) received comparable surgeries, but bilateral guide cannulae were implanted in the shell/core border of the nucleus accumbens (NAc; +1.5 mm AP; ±1.2 mm ML; –5.4 mm DV).

### Baclofen/muscimol infusions

Rats were bilaterally injected with 0.3 μl of either artificial CSF (aCSF) or a 1.0 nmol/0.1 nmol mixture of the GABA-A and GABA-B receptor agonists baclofen and muscimol (BM; Tocris Bioscience) dissolved in aCSF. Injection cannulae (33 gauge, Plastics One) were inserted bilaterally and protruded 1 mm below the guide cannulae. Solutions were delivered over the course of 1 min using a microinfusion pump (UMP3/Micro 4, World Precision Instruments), and the injection cannulae were maintained in place for an extra minute to allow diffusion of the fluid. For the NAc locomotion task, injection cannulae extended 2 mm beyond guide cannulae. Rats were tested at least 5 min after the injection cannulae were removed.

### Apparatus

All operant testing was conducted in chambers housed in sound attenuation cubicles (Med Associates). Nose pokes were located on the left and right walls of the operant chambers. Beneath the right nose poke was a well where reward (0.1 ml of 15% sucrose solution) was dispensed. Each chamber was illuminated by a house light, and a fan provided ∼60-dBA background noise. The same boxes were used for extinction, cue-induced reinstatement, and PR experiments, although the inactive nose poke was inaccessible during extinction sessions. For the NAc locomotion experiments, rats were placed in a Plexiglas chamber (39.4 × 39.4 × 52.1 cm) with black colored walls and a stainless-steel grid floor. A digital camcorder (Canon VIXIA HF R52) was mounted above the box to record locomotor activity.

### Behavioral test groups

Three operant test groups were used in these studies. The first group received inactivation during fixed ratio 1 (FR1) sucrose seeking. The second group received inactivation during early and late extinction. The third group received inactivation during cue-induced reinstatement and PR sessions. The FR1 group received bilateral and unilateral inactivation. Because no major effects were found with unilateral inactivation, the extinction and cue-induced reinstatement/PR groups received only bilateral inactivation. The FR1 group also received inactivation during extinction, cue-induced reinstatement, and PR. In this group, we observed no significant effects of manipulation in any of these tests, leading us to consider the possibility that multiple infusions during self-administration resulted in long-lasting damage occluding any potential effects of regional inactivation. Thus, separate groups were run for extinction and cue-induced reinstatement/PR sessions.

### Sucrose self-administration

**Figure 1.**
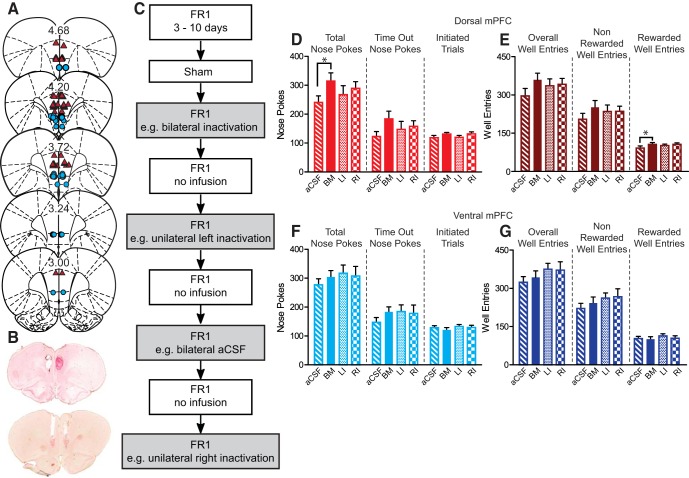
Cannula placements, test design, and FR1 data. ***A***, Cannula placements for FR1 cohort. Dorsal mPFC cannula placements (triangles) and ventral mPFC cannula placements (circles). Numbers are A/P distance from bregma. ***B***, Histology of coronal slices stained with neutral red showing cannula tracks for dorsal (top) and ventral (bottom) mPFC. ***C***, Timeline for FR1 testing. Rats were retrained for 3–10 d after surgery. They then received sham infusions followed by 8 d of FR1 tests. Rats received one of four infusions every other day of testing: bilateral inactivation, bilateral aCSF, unilateral left, or right inactivation, counterbalanced across rats. All rats received all four conditions. aCSF (stripes) = control infusion, BI (solid) = bilateral inactivation, LI (dots) = inactivation of left hemisphere, RI (checkers) = inactivation of right hemisphere. ***D***, ***F***, Total number of nose pokes, time-out nose pokes, and initiated trials. ***E***, ***G***, Total number of well entries, non-rewarded well entries, and rewarded well entries. ***D***, ***E***, There was a significant increase in total number of nose pokes and total number of rewarded well entries when the dorsal mPFC was bilaterally inactivated (*). ***F***, ***G***, Ventral mPFC inactivation did not affect nose poking or well entries; *p < 0.05, Dunnett’s test for planned multiple comparison.

Before surgery, rats were trained to self-administer sucrose on a FR1 schedule. A 10- to 15-s house light illumination signaled the time-out, during which nose poking in the left (inactive) and right (active) nose pokes were recorded but did not elicit any consequences. On house light offset, nose poking in the right nose poke elicited a tone (15 kHz, 74 dBA, 1 s) and delivery of 0.1-ml 15% sucrose in the well beneath the nose poke. The first active poke after the time-out was counted as a “trial initiation” to distinguish these pokes from other (e.g., time-out) active nose pokes. Trials in which the rat exited the nose poke and entered the well in <1 s after sucrose was dispensed were counted as “rewarded well-entries”. Surgeries were performed after rats reached at least 100 rewarded well-entries and met criteria of 80% rewards collected within 1 s of delivery. After recovery, rats were retrained for 3–10 d (Fig. 1*C*). After re-training, rats received a sham infusion in which the injector cannula was inserted and left in place for 1 min, but nothing was infused. Testing started the following day. Rats were tested on an FR1 schedule for 8 d in total after sham infusion test day. Sessions lasted 1 h or until the rat performed 160 trials. During testing, each rat received four separate infusions in counterbalanced order across days: (1) bilateral BM, (2) bilateral aCSF, (3) BM in the left hemisphere and aCSF in the right hemisphere, and (4) aCSF in the right hemisphere and BM in the left hemisphere (Fig. 1*C*). In between infusion days, rats were run on FR1 with no infusion to avoid potential rebound effects and to maintain task performance.

### Extinction

**Figure 2.**
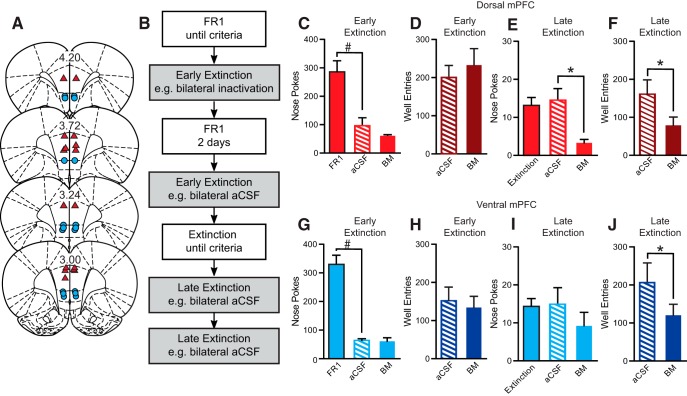
Cannula placements, test design, and extinction data for extinction cohort. ***A***, Dorsal mPFC cannula placements (triangles) and ventral mPFC cannula placements (circles). ***B***, Timeline for extinction task. Extinction rats were trained on FR1 but only received bilateral infusions during early and late extinction. ***C***, ***G***, There was a significant decrease in number of nose pokes between last day of FR1 and aCSF treatment during extinction (#). ***C***, ***D***, ***G***, ***H***, Bilateral inactivation of dorsal or ventral mPFC did not significantly affect nose pokes or well entries during early extinction. ***E***, ***F***, Inactivation of dorsal mPFC during late extinction decreased nose pokes and well entries (*). ***I***, There was no effect of ventral mPFC inactivation for number of nose pokes during late extinction. ***J***, However, there was a decrease in number of well entries during ventral mPFC inactivation during late extinction (*). # and *p < 0.05, paired *t* test.

A second cohort of rats was trained to reliably respond for sucrose under the FR1 schedule described above. After stable FR1 performance (100 rewarded well-entries and 80% rewards collected within 1 s), rats received inactivation tests during early and late extinction sessions (Fig. 2*B*). Rats received one of two conditions on the first day of early extinction: BM or aCSF. They were then retrained on FR1 for 2 d, and received a second day of early extinction during infusion with the opposite drug or vehicle combination. We included 2 d of FR1 retraining in between each early extinction day to allow paired analysis of early extinction within rats. Rats were then extinguished until they responded with fewer than 20 nose pokes per session for two continuous sessions. On the last 2 d of extinction (late extinction) rats received counterbalanced BM/aCSF treatments as in early extinction.

### Cue-induced reinstatement

**Figure 3.**
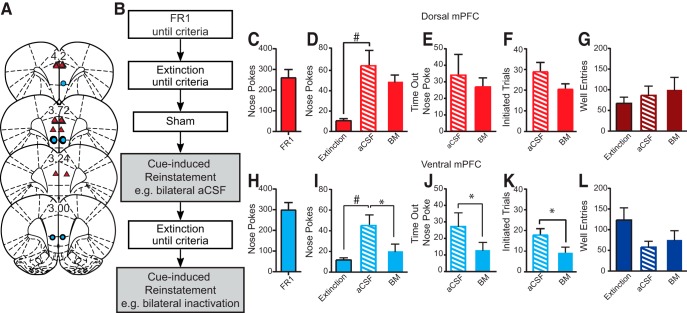
Cannula placements, test design, and reinstatement data for reinstatement cohort. ***A***, Dorsal mPFC cannula placements (triangles) and ventral mPFC cannula placements (circles). ***B***, Timeline for reinstatement task. Reinstatement rats were trained on FR1 and extinction but only received bilateral infusion during reinstatement. ***C***, ***H***, Number of nose pokes during FR1 session the day before extinction training. ***D***, ***I***, There was a significant increase in nose pokes on aCSF reinstatement infusion day compared to last day of extinction (#). ***D–G***, Bilateral inactivation of dorsal mPFC did not significantly affect nose pokes, time-out nose pokes, initiated trials, or well entries. ***I–L***, Bilateral ventral mPFC inactivation significantly decreased total number of nose pokes, time out nose pokes, and initiated trials (*), but not rewarded well entries; # and *p < 0.05, paired *t* test.

A third cohort of rats was trained to reliably respond for sucrose under the FR1 schedule described above and then extinguished to the point of responding with fewer than 20 nose pokes per session for two continuous sessions (Fig. 3*B*). Rats were then tested in cue-induced reinstatement sessions. During reinstatement, nose pokes on an FR1 schedule elicited a tone but no sucrose delivery. Rats were bilaterally infused with either BM or aCSF on two separate reinstatement days in a counterbalanced fashion. Reinstatement tests were separated by extinction sessions until rats reached criteria of two sessions with fewer than 20 nose pokes.

### Progressive ratio

After cue-induced reinstatement, the same rats that were tested on reinstatement were tested on a PR sucrose seeking task. The PR test environment was the same as for FR1, but the number of nose pokes required to receive reward increased on each trial based on the equation: response ratio (rounded to the nearest integer) = [5e^(injection number × 0.2)^] – 5 (Richardson and Roberts, 1996). The highest reward number acquired was considered the breakpoint and was analyzed, along with nose pokes and well entries, as a measure of motivation. Rats were bilaterally infused with BM and aCSF before testing on separate PR testing days. PR testing lasted either 6 h or until 60 min of no nose pokes occurred. PR test days were separated by two consecutive days of FR1 training.

### Spontaneous locomotion

To verify the behavioral effects of BM, we tested the effect of NAc inactivation during a spontaneous locomotor assay. Methods were based on those described previously (Fuchs et al., 2004). A new cohort of rats was infused with either BM or aCSF in NAc and placed into a novel box 10 min after the infusion. Behavior was video recorded for 1 h and later analyzed using ANY-maze software (ANY-maze), in which we divided the chamber in 8 zones and counted numbers of line crosses into each zone.

### Whole-cell patch-clamp

To verify the physiologic effects of BM, we recorded the activity of mPFC neurons *in vitro* during bath application of BM. Seven neurons from two male Long–Evans rats, ∼25 d old, were included in this analysis. Rats were deeply anesthetized with isoflurane and sacrificed using rapid decapitation, and brains were removed and immersed in ice-cold cutting solution (250 mM glycerol, 26 mM NaHCO_3_, 2.5 mM KCl, 1.2 mM NaH_2_PO_4_, 11 mM glucose, 2.4 mM CaCl_2_, and 1.2 mM MgCl_2_; 310 mOsm; pH 7.4 when saturated with 95% O_2_/5% CO_2_); 300-μm coronal sections were obtained using a vibrating blade microtome (VT1000S, Leica Biosystems Inc.), and were immediately transferred to aCSF (37°C; 250 mM glycerol, 26 mM NaHCO_3_, 2.5 mM KCl, 1.2 mM NaH_2_PO_4_, 11 mM glucose, 2.4 mM CaCl_2_, and 1.2 mM MgCl_2_; 310 mOsm; pH 7.4 when saturated with 95% O_2_/5% CO_2_). After 30 min under these conditions, slices were kept in bubbled aCSF at room temperature for the remainder of the experiment. Glass pipettes were pulled from borosilicate glass tubes (1B150F-4, World Precision Instruments) using a two-stage, vertical puller (PC-10, Narishige International USA), and were backfilled with internal solution (110 mM K-gluconate, 8 mM NaCl, 30 mM KCl, 1 mM MgCl_2_, 10 mM HEPES, 0.2 mM EGTA, 2 mM Mg-ATP, 0.5 mM GTP; 298 mOsm; pH 7.4). When filled, pipettes had a tip resistance of 5–8 MΩ. Once whole-cell configuration was achieved, cells were allowed to stabilize for at least 5 min before recordings proceeded. Spontaneous postsynaptic currents (sPSCs) were recorded in voltage clamp mode from pyramidal neurons held at –70 mV in the medial wall of the PFC. Recordings were taken before (range: 3–11 min), during (range: 3–13 min), and after (range: 4–30 min) application of BM. Series resistance was monitored throughout the recordings. Recordings were concatenated offline in Igor Pro (Wavemetrics) to create one contiguous file, which was then exported to Spike2 (Cambridge Electronic Design Limited) where it was low-pass filtered above 100 Hz. Timestamps were obtained in Spike2 through wave form-based template matching. For both the pretreatment and treatment segments, the length of each recording was standardized to that of the shortest recording by exclusively including the last 3 min, and PSC frequency was tabulated for 3-min periods before, during, and after BM treatment.

### Histology

After final test sessions, rats were deeply anesthetized with ketamine/xylazine (80:10 mg/kg, i.p.), and brains were extracted, stored in 4% paraformaldehyde overnight, and transferred to 20% (wt/vol) solution of sucrose/0.1% sodium azide in phosphate buffer at 4°C. Coronal sections 40 μm thick were cut using a cryostat, mounted on slides, stained with neutral red and cover slipped. Cannula placements were verified by comparing cannula damage to a rat brain atlas (Paxinos and Watson, 2007). Two ventral mPFC rats in the FR1 group, one ventral mPFC rat in the extinction group, and one dorsal and one ventral mPFC rat in the reinstatement group were excluded from analysis due to blocked cannulae or excessive tissue damage. Two rats were excluded from the locomotion task because of cannula misplacements. Cannula placements for each group are shown in the behavior-associated figures.

### Analysis

Data were analyzed using Prism (GraphPad Software). Total numbers and rates (total number divided by the time taken to complete the task) of active and inactive nose pokes and well entries for the FR1 task were calculated and differences were assessed using one-way repeated measures (RM) ANOVA followed by planned Dunnett’s test for multiple comparisons to compare each treatment to bilateral aCSF. In addition to number of responses, we also measured response rate during FR1 as some rats finished the task before the 1 h of duration of the task. Total numbers of nose pokes and well entries for extinction, cue-induced reinstatement, and PR data were analyzed using one-way ANOVA and paired *t* tests. Numbers of nose pokes during FR1, early extinction, late extinction, and cue-induced reinstatement were divided into quartiles and data were analyzed using paired two-way ANOVA (treatment × quartile). Locomotion was analyzed using a two-way ANOVA comparing an interaction between 10-min bins of time and infusion condition. Simple effects for locomotion data, as well as patch clamp data were analyzed using a one-way RM ANOVA. Means and SEM are presented as mean ± SEM.

## Results

### Dorsal, but not ventral, mPFC inactivation increased reward seeking during FR1 sucrose self-administration

All rats were highly motivated to perform the FR1 sucrose seeking task (Fig. 1). RM ANOVA did not reveal significant differences among groups for number of nose pokes (*F*_(3,19)_ = 2.37, *p* = 0.08). However, planned Dunnet’s tests versus aCSF revealed an increase in total number of nose pokes when dorsal mPFC was bilaterally inactivated (*p* < 0.05, Dunnett’s; Fig. 1*D*). Bilateral inactivation also increased overall rate of nose pokes (*F*_(3,19)_ = 2.76, *p* = 0.050, RM ANOVA across all manipulations; *p* < 0.05, Dunnett’s for bilateral BM vs bilateral aCSF) , and in rate of time out nose pokes (*F*_(3,19)_ = 2.31, *p* = 0.086, RM ANOVA; *p* < 0.05 Dunnett’s). Bilateral dorsal mPFC inactivation increased number of rewarded well entries, defined as entering the well in <1 s after sucrose was dispensed, compared to aCSF (*F*_(3,19)_ = 2.40, *p* = 0.077, RM ANOVA; *p* < 0.05 Dunnett’s; Fig. 1*E*). We also observed a significant increase in the number of initiated trials (*F*_(3,19)_ = 3.13, *p* = 0.033), but Dunnett’s tests did not reveal any significant differences compared to bilateral aCSF (*p* > 0.05). Unilateral inactivation of dorsal mPFC had no significant effect on numbers or rate of nose pokes or well entries (all *p* > 0.05, Dunnett’s). Ventral mPFC inactivation, bilateral or unilateral, had no significant effects on number or rate of nose pokes or well entries (all *p* > 0.05, RM ANOVA and Dunnett’s; Fig. 1*F*,*G*). There were also no effects of inactivation on latency to initiate trials or collect reward after dorsal or ventral mPFC inactivation (all *p* > 0.05, RM ANOVA and Dunnett’s). Inactive nose poke responses were low and there were no effects of manipulation on inactive responses (range means 1.6–5.3, all *p* > 0.05, RM ANOVA and Dunnett’s)

### Dorsal and ventral mPFC inactivation decreased reward seeking during extinction

Fifteen rats received bilateral inactivation of dorsal (*n* = 8) or ventral (*n* = 7) mPFC during early (days 1 and 2) and late (2 d of <20 nose pokes) extinction sessions (Fig. 2). There were no effects of inactivation of dorsal or ventral mPFC during early extinction. However, inactivation of dorsal mPFC significantly reduced both nose pokes (*t*_(7)_ = 4.00, *p* = 0.005) and well entries (*t*_(7)_ = 2.38, *p* = 0.049) (Fig. 2*E*,*F*). Inactivation of ventral mPFC significantly decreased well entries (*t*_(6)_ = 2.86, *p* = 0.029) (Fig. 2*J*) and, although it appeared that nose pokes were reduced (Fig. 2*I*), this effect was not significant (*t*_(6)_ = 1.01, *p* = 0.35).

### Ventral, but not dorsal, mPFC inactivation decreased reward seeking during cue-induced reinstatement

After aCSF treatment on cue-induced reinstatement tests, rats exhibited a significantly increased number of nose pokes compared to the last day of extinction [dorsal mPFC (Fig. 3*D*); *t*_(6)_ = 3.44, *p* = 0.014; ventral mPFC (Fig. 3*I*); *t*_(6)_ = 3.88, *p* = 0.008, paired *t* test). Bilateral inactivation of ventral mPFC significantly decreased total number of reinstatement nose pokes (*t*_(6)_ = 3.05, *p* = 0.023, paired *t* test; Fig. 3*I*) relative to aCSF treatment. There was also a decrease in number of time-out nose pokes (*t*_(6)_ = 2.57, *p* = 0.042; paired *t* test; Fig. 3*J*) and number of initiated trials (*t*_(6)_ = 3.71, *p* = 0.010; Fig. 3*K*). There were no significant effects of bilateral inactivation of dorsal mPFC on nose pokes or well entries (all *p* > 0.05, paired *t* test; Fig. 3*D–G*). There were also no significant effects of either dorsal or ventral mPFC inactivation on inactive nose pokes (all *p* > 0.05, paired *t* test). Of note the effects on ventral mPFC inactivation observed here were directionally consistent with those observed during reinstatement in our first test group (see Materials and Methods). Although the effects in that group were milder and not significant (likely due to eight prior cannula infusions), the directional consistency across study groups combined with the significant effects observed here supports the reliability of these findings.

### Neither dorsal or ventral mPFC inactivation affected reward seeking during PR sucrose self-administration

**Figure 4.**
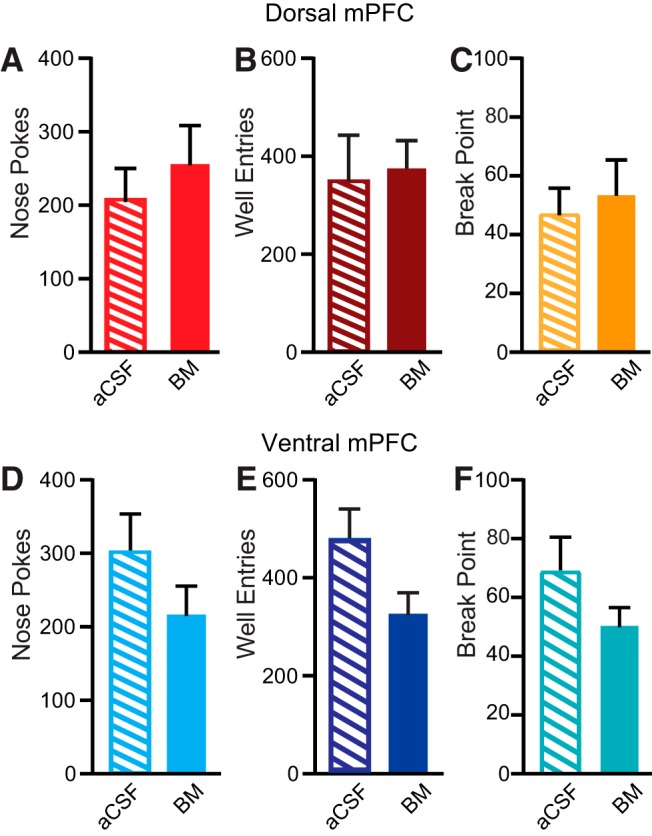
PR data. No significant effects of dorsal mPFC (***A–C***) or ventral mPFC (***D–F***) inactivation on nose pokes, well entries, or break point.

Rats demonstrated reliably high levels of sucrose seeking during PR as measured by nose pokes, well entries, and breakpoints (Fig. 4). There was no effect of either dorsal or ventral mPFC inactivation on numbers of total active nose pokes, initiated trials, time-out nose pokes, well entries, breakpoint values, or inactive nose pokes (all *p* > 0.05, paired *t* tests).

### Within-session analysis of inactivation effects

**Figure 5.**
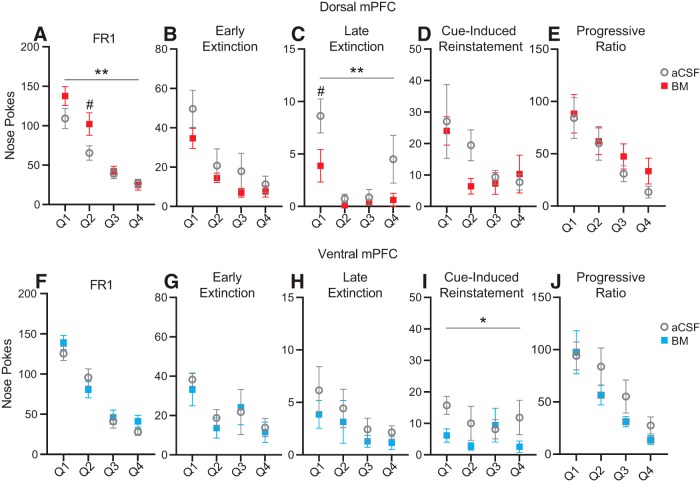
Average number of nose pokes per quartile for FR1 (***A***, ***F***), early extinction (***B***, ***G***), late extinction (***C***, ***H***), cue-induced reinstatement (***D***, ***I***), and PR (***E***, ***J***) for during inactivation of dosal mPFC (***A–E***) or ventral mPFC (***F–J***). Dorsal mPFC inactivation increased FR1 nose pokes, notably in the first half of the session. Dorsal mPFC inactivation decreased late extinction nose pokes, primarily early in the session. Ventral mPFC inactivation decreased cue-induced reinstatement nose pokes, but the effect was distributed across the session; *p < 0.05, **p < 0.01, two-factor ANOVA (treatment × quartile); #*p* < 0.05, Sidak’s MCT.

One possible outcome of inactivation may have been a transient effect during part of the session that was not overall apparent by comparing total numbers of nose pokes (e.g., effects only early or late during a session). To address this, we divided sessions into four quartiles and compared nose poking during BM versus aCSF sessions using a RM two-factor ANOVA (treatment × quartile). The results of these analyses are shown in Figure 5 for FR1, early and late extinction, and cue-induced reinstatement. Analyses were performed for PR as well, but there were no significant effects either overall or within sessions. As expected there were overall significant main effects of treatment for dorsal mPFC inactivation during FR1 (*F*_(1,76)_ = 7.71, *p* = 0.007) and late extinction (*F*_(1,28)_ = 9.27, *p* = 0.005). *Post hoc* multiple comparisons (Sidak’s MCT) revealed significant differences during the second quartile during FR1 (*t* = 3.11, *p* = 0.011) and during the first quartile during late extinction (*t* = 2.97, *p* = 0.024). Despite a significant main effect of treatment after ventral mPFC inactivation during cue-induced reinstatement (*F*_(1,24)_ = 5.22, *p* = 0.030), there were no significant treatment effects in any quartile, indicating consistent small reductions throughout the entire session. There were no effects of treatment on nose poking behavior in any of the other analyzed sessions and no interaction effects.

### Baclofen/muscimol infusions into the NAc disrupted spontaneous locomotion

**Figure 6.**
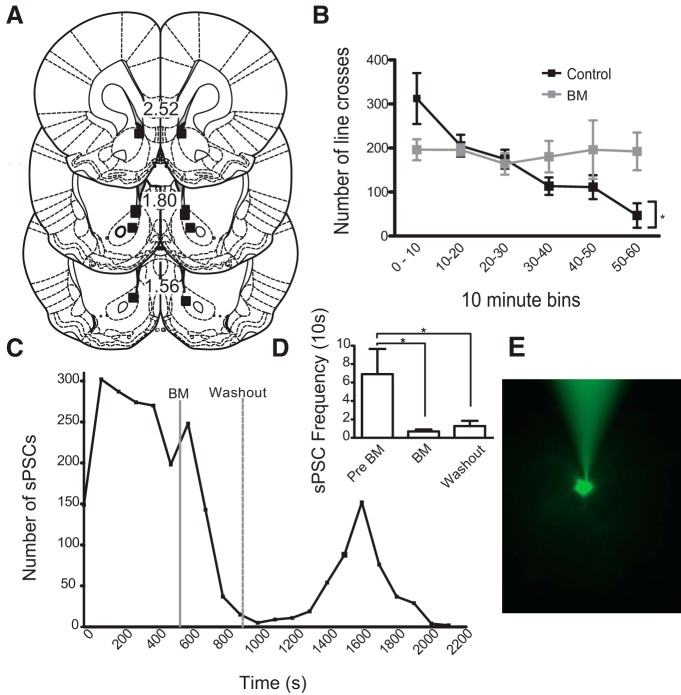
Behavioral and physiological verification of BM efficacy. BM infusion in NAc disrupted spontaneous locomotion, and *in vitro* BM infusion decreased sPSCs in mPFC neurons. ***A***, Cannula placements for locomotion study. ***B***, aCSF-infused rats decreased locomotion over time, but this effect was not observed for rats receiving BM infusions; *p < 0.05, RM ANOVA. ***C***, sPSCs of one representative neuron. ***D***, Mean sPSC frequency before BM, after BM, and after washout; *p < 0.05, Tukey’s multiple comparison test. ***E***, Example recorded rat mPFC neuron stained with Alexa Fluor 488.

Because mPFC inactivation results were unexpected compared to previous studies, we verified the effect of our BM infusions by inactivating NAc during spontaneous locomotion - a reliable behavioral assay that is sensitive to BM inactivation of NAc (Fuchs et al., 2004; Stopper and Floresco, 2011). We infused BM or aCSF bilaterally in NAc (Fig. 6*A*) and measured locomotor activity in 10 min bins (Fig. 6*B*). As expected, there was a statistically significant interaction between the effects of drug and time on locomotion (*F*_(5,24)_ = 3.35, *p* = 0.020; two-way ANOVA; Fig. 6*B*). Locomotion was initially elevated and decreased over time in aCSF-infused rats (*F*_(5,2)_ = 6.99, *p* = 0.005; one-way ANOVA). BM-infused rats showed decreased locomotion during the initial stages of testing relative to aCSF and did not show a significant difference in locomotion over time (*F*_(5,2)_ = 0.22, *p* = 0.947; one-way ANOVA). These results are consistent with previous findings (Fuchs et al., 2004; Stopper and Floresco, 2011), and confirmed that differences observed between our mPFC inactivation effects and those described in previous studies were not due to lack of efficacy of our BM infusions.

### Baclofen/muscimol decreased sPSCs in rat prefrontal neurons

To further validate the inhibitory influence of our BM infusions at the specific concentrations given, we measured the effects of BM application on mPFC neuronal activity *in vitro*. BM bath application significantly decreased spontaneous activity in prefrontal neurons (*n* = 7 neurons from two rats; Fig. 6*C*), as demonstrated by a statistically significant suppressive effect of BM on sPSCs (5b; *F*_(2,6)_ = 5.6, *p* = 0.019; one-way ANOVA). *Post hoc* analyses revealed a significant decrease in number of sPSCs during BM and during washout (*p* < 0.05; Tukey’s multiple comparison test; Fig. 6*D*). These results confirm the reliably inhibitory effect on mPFC neurons of the BM cocktail concentration used in our behavioral studies.

## Discussion

Previous work has led to the hypothesis that dorsal and ventral mPFC play opposing roles in driving behavior, particularly in the context of action execution versus suppression (Peters et al., 2009; Gass and Chandler, 2013; Barker et al., 2014; Gourley and Taylor, 2016; Muller Ewald and LaLumiere, 2018). The reasons for this distinction are relatively clear, as described in multiple studies referenced in detail in Peters et al. (2009), Moorman et al. (2015), Gourley and Taylor (2016), and Muller Ewald and LaLumiere (2018). For example, manipulation of dorsal mPFC frequently disrupts behavioral execution such as drug/reward seeking or expression of conditioned fear (McFarland et al., 2004; Ishikawa et al., 2008b; Sierra-Mercado et al., 2011; Eddy et al., 2016; Trask et al., 2017). In contrast, ventral mPFC manipulation has been shown to regulate behavioral inhibition in certain circumstances, such as during extinction (Ishikawa et al., 2008b; Peters et al., 2008; Sierra-Mercado et al., 2011; Peters and De Vries, 2013; Augur et al., 2016; Muller Ewald and LaLumiere, 2018). However, a number of studies have called the ubiquity of this dichotomy into question (McFarland et al., 2003; Jonkman et al., 2009; Bossert et al., 2011; Chen et al., 2013; Willcocks and McNally, 2013; Martín-García et al., 2014; Moorman and Aston-Jones, 2015; Moorman et al., 2015; McGlinchey et al., 2016; Gutman et al., 2017a), prompting us to perform the experiments described here.

Our results do not support a clear dichotomy for dorsal versus ventral mPFC during natural reward seeking. Based on the studies described above, we expected that inactivation of dorsal mPFC would decrease sucrose seeking and have no effect on extinction, and that ventral mPFC inactivation would increase sucrose seeking and induce reinstatement during extinction. Instead, dorsal mPFC inactivation increased sucrose seeking during FR1 self-administration and had no effect during cue-induced reinstatement. Ventral mPFC inactivation decreased sucrose seeking during cue-induced reinstatement and had no effect during FR1. Inactivation of both subregions decreased responding during late extinction, as shown by significantly reduced nose pokes and well-entries after dorsal mPFC inactivation and significantly reduced well entries after ventral mPFC inactivation. Inhibition of neither region influenced reward seeking under a PR schedule, again in line with a lack of general regulation of action execution or suppression. Together, these results make a strong case against a universal dichotomous role for dorsal versus ventral mPFC in action execution versus inhibition.

Because our results were somewhat surprising, we performed controls to verify that our inactivations were effective. NAc inactivation with BM decreased spontaneous locomotion, in line with previous work (Fuchs et al., 2004; Stopper and Floresco, 2011), and bath application of BM inhibited spontaneous activity in rat mPFC neurons. Both findings support the efficacy of our BM treatments. We conclude that the effects observed did in fact result from mPFC inactivation during behavior.

The absence of absolute differences is in line with some previous work examining dorsal versus ventral mPFC in execution versus suppression of reward seeking, as described above. However, in many of these studies, the tasks employed used slightly more complex rules to guide behavior such as the use of a discriminative stimulus (Ishikawa et al., 2008b; Moorman and Aston-Jones, 2015; Gutman et al., 2017a). The goal of this study was to attempt to isolate self-initiated action execution or inhibition to identify mPFC subregion contributions, in line with those seen in studies of drug seeking. If, in fact, dorsal and ventral mPFC play opposing roles in the regulation of action execution and inhibition, this should have been clearly demonstrable under the behavioral conditions in the current study. Instead, our data argue for an influence of context, in this case the behavioral task performed, on mPFC regulation of behavior, as reported previously (Moorman and Aston-Jones, 2015; McGlinchey et al., 2016). Similarly complex results have been observed in Pavlovian contexts (Sangha et al., 2014; Mendoza et al., 2015).

An additional finding was an overall lack of effect of unilateral inactivation on sucrose seeking. Previous studies have shown differential contributions of left versus right mPFC in stress-related paradigms (Sullivan and Gratton, 2002a), leading us to consider the possibility that left or right mPFC may play a disproportionate role in reward seeking. Although the only significant effect during FR1 was seen after bilateral dorsal mPFC inactivation, right hemisphere dorsal mPFC inhibition produced qualitatively similar results in some cases, although the effects were not significant in planned comparisons. Accordingly, we did not pursue unilateral inactivations in cue-induced reinstatement or PR. Despite our overall lack of lateralization findings, a study more directly designed to explore this question may be worth undertaking in future work.

One possible distinction between our results and some previous studies is the type of behavior used to evaluate mPFC control. It might not be surprising that studies using different behaviors may result in different effects of mPFC inactivation. This is most obvious for fear conditioning studies, where the behavioral readout is actually freezing – a combination of both an emitted behavior (based on a decision to freeze) and a lack of action (freezing), in some cases combined with a suppression of food self-administration (Sierra-Mercado et al., 2011; Giustino and Maren, 2015). A more subtle distinction is between the use of nose poke operanda, as employed here and in some studies (Willcocks and McNally, 2013), and the use of lever presses in other previous studies (Ishikawa et al., 2008b; Peters et al., 2008). Although this may not be a critical determinant, there are differential learning rates between nose poke and lever presses (Schindler et al., 1993), and different neural substrates underlying the two behaviors (Bassareo et al., 2015). This influence of action type on mPFC contributions to behavior is currently under investigation in our laboratory.

The most salient differences exist between our findings and previous studies of cocaine self-administration, extinction, and reinstatement. Multiple studies have shown a prominent role for dorsal mPFC in driving cue-induced reinstatement of cocaine seeking as well as a critical role for ventral mPFC suppressing cocaine seeking after extinction learning (McFarland and Kalivas, 2001; McLaughlin and See, 2003; Fuchs et al., 2005; LaLumiere and Kalivas, 2008; Peters et al., 2008, 2009; Gass and Chandler, 2013; Moorman et al., 2015; Gourley and Taylor, 2016; Muller Ewald and LaLumiere, 2018), although see counterexamples such as Chen et al. (2013) and others described in Moorman et al. (2015). A fundamental and yet-unanswered question is why these reliable roles for dorsal and ventral mPFC in regulation of cocaine-associated actions are not observed in sucrose seeking, as described here, or in other types of reward seeking (McFarland and Kalivas, 2001; McGlinchey et al., 2016; Gutman et al., 2017b). One possibility might be the nature of the reinforcer. Cocaine may be a more salient reinforcer than sucrose, thereby differentially engaging mPFC subregions based on some motivational intensity gradient, although see Lenoir et al. (2007). Another possible explanation is that repeated cocaine induces neuroplastic changes in the mPFC that results in differential regulation of seeking behavior relative to natural rewards (Robinson and Kolb, 1999; Robinson et al., 2001; McFarland et al., 2003; Muñoz-Cuevas et al., 2013; Radley et al., 2015; Siemsen et al., 2019). Cocaine also induces both appetitive and aversive behaviors (Ettenberg, 2004), whereas there are fewer aversive components to sucrose. mPFC subregions may regulate behaviors associated with a mixed-valence pharmacological stimulus differently than a purely appetitive reinforcer. Another potential explanation may be the way that reward is delivered: cocaine is typically self-administered intravenously whereas sucrose must be collected following a correct operant response. These and other potential explanations are currently under investigation in our laboratory, motivated by the very clear differences in mPFC contributions to ostensibly the same behavior related to different outcomes.

Rodent mPFC subregions play a host of functions instead of or in addition to action expression versus inhibition (Dalley et al., 2004; Kesner and Churchwell, 2011; Euston et al., 2012; Cassaday et al., 2014). In some cases, dorsal and ventral mPFC functions have been shown to be dichotomous. For example, when comparing goal-directed (outcome sensitive) versus habitual (outcome insensitive) reward seeking, there appear to be differences whereby dorsal mPFC preferentially regulates goal-directed and ventral mPFC controls habitual behaviors (Killcross and Coutureau, 2003; Smith et al., 2012; Barker et al., 2014, 2015; Smith and Laiks, 2018). Because we did not explicitly test goal-directed versus habitual behavior using, e.g., reward devaluation, we cannot make strong claims about our effects in this framework, although this might be a useful avenue for future studies integrating mPFC functions across behavioral paradigms.

Despite not observing clear dichotomous dorsal and ventral mPFC functions, we did see selective effects of inactivation. Bilateral dorsal mPFC inactivation increased FR1 sucrose seeking. This finding is aligned with those demonstrating a response-suppression role for dorsal mPFC, such as is observed during punishment-associated cocaine seeking (Chen et al., 2013). It is also in line with previous work demonstrating increased operant behavior following dorsal mPFC inactivation (Jonkman et al., 2009) and other studies showing dorsal mPFC involvement in response inhibition in other tasks (Narayanan et al., 2006; Ragozzino, 2007; MacLeod and Bucci, 2010; Bari and Robbins, 2013; Meyer and Bucci, 2014; Hardung et al., 2017). Although in our study there was no need for dorsal mPFC to suppress behavior, reward-associated decisions, even without challenges such as punishment, may require balance between response inhibition driven by the effort associated with reward seeking versus the excitatory drive to acquire a reward. Here, dorsal mPFC inactivation increased both rewarded and non-rewarded nose pokes. On the one hand, this suggests that dorsal mPFC inactivation resulted in a general release on any inhibition of behavior, or “taking the brakes off.” However, it is worth noting that these increases were not seen for inactive nose pokes, during other non-rewarded tasks (extinction, reinstatement), or even during PR testing, in which rewards were available. In fact, dorsal mPFC inactivation decreased nose pokes in late extinction, when reward was not available. These results underscore the fact that behavioral context and task details influence contributions of mPFC to behavior, in some cases dorsal mPFC plays a response-invigorating role whereas in others it is suppressive.

Similarly, ventral mPFC is frequently associated with behavior suppression, particularly during extinction (Maren and Quirk, 2004; Peters et al., 2009; Sierra-Mercado et al., 2011; Gourley and Taylor, 2016; Muller Ewald and LaLumiere, 2018). In our study, ventral mPFC inactivation decreased cue-induced reinstatement, in line with previous studies of reinstatement for heroin (Rogers et al., 2008; Bossert et al., 2011, 2012) and methamphetamine (Rocha and Kalivas, 2010) seeking, but in contrast with previous studies of cocaine seeking and fear conditioning (LaLumiere and Kalivas, 2008; Peters et al., 2008; Muller Ewald and LaLumiere, 2018). Ventral mPFC inactivation also had little inhibitory effect on alcohol seeking and did not counteract extinction (Willcocks and McNally, 2013). It is unclear what differentiates ventral mPFC contributions to sucrose, alcohol, methamphetamine, and heroin reinstatement versus extinction of cocaine and fear conditioning, although there are obviously substantial differences in neural encoding of different drugs/rewards/punishment, type of reinstatement (e.g., cue vs context), or other as-yet undefined factors (Badiani et al., 2011; Peters et al., 2013).

In summary, our results make it clear that dorsal and ventral mPFC do not universally exhibit opposing control over behavior. Instead our findings should be integrated with previous work in which dichotomies were observed, along with other studies involving, e.g., response inhibition, to identify how different behavioral tasks differentially engage mPFC subregions. We also note that a focus on neuronal ensembles and networks should be emphasized in future work (Gabbott et al., 2005; Bossert et al., 2011; Moorman et al., 2015; Pfarr et al., 2015; Warren et al., 2016; George and Hope, 2017; Kim et al., 2017). It is possible that different findings across studies may result from differentially targeting subregional circuits (e.g., mPFC projections to NAc core, shell, or amygdala). The use of circuit specific techniques and other precision-enhancing technologies, combined with a rigorous assessment of behavioral details, has the potential to significantly advance our understanding of mPFC function, including its contributions to complex behavior and mental diseases.

## Synthesis

Reviewing Editor: Mark Laubach, American University Decisions are customarily a result of the Reviewing Editor and the peer reviewers coming together and discussing their recommendations until a consensus is reached. When revisions are invited, a fact-based synthesis statement explaining their decision and outlining what is needed to prepare a revision will be listed below. The following reviewer(s) agreed to reveal their identity: Nathan Marchant, Matthew Roesch. Note: If this manuscript was transferred from JNeurosci and a decision was made to accept the manuscript without peer review, a brief statement to this effect will instead be what is listed below.

Both reviewers indicated decisions of Accept: As is.

Reviewer #1:

I believe that this manuscript is substantially improved from the original. I appreciate the additional experimental groups, and the within-session analysis adds to the story.

The findings presented here are of interest to the field, and highlight the complexity of mPFC function, which is often overlooked when others try to summarize this function within a go/stop dichotomy type of explanation.

Reviewer #2:

I thought the authors did an excellent job reviewing of addressing my concerns. I agree that they offer a clear counterpoint to the go/stop hypothesis and look forward to future work from their lab that will define a new model/framework.
